# Supplementary figures and images for: Lung image segmentation with improved U-Net, V-Net and Seg-Net techniques
Source: PeerJ Comput Sci. 2025 Feb 13;11:e2700. doi: 10.7717/peerj-cs.2700 (PMC11888921; doi:10.7717/peerj-cs.2700)

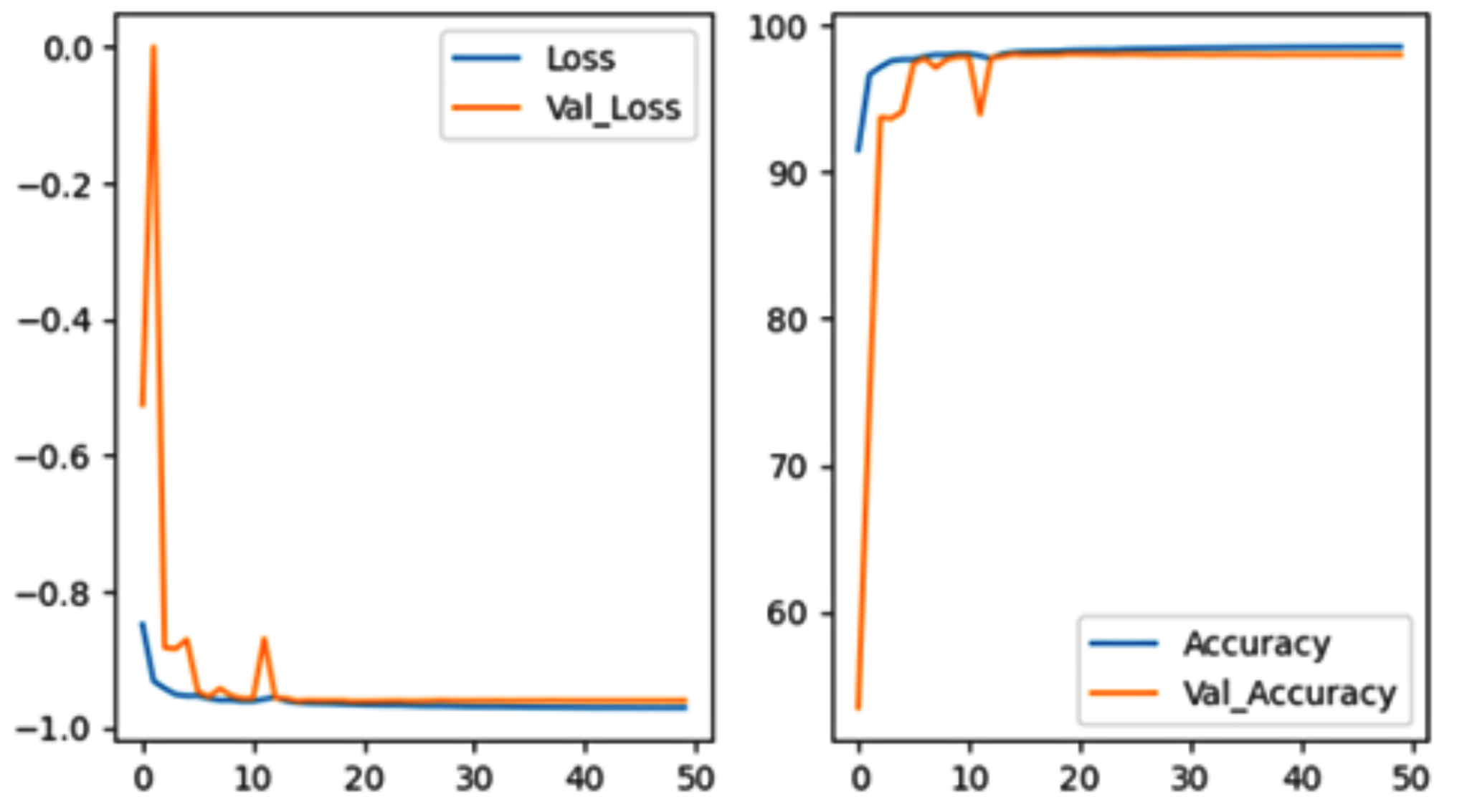

Supplement: Supplemental Information 6 [file peerj-cs-11-2700-s006.png]

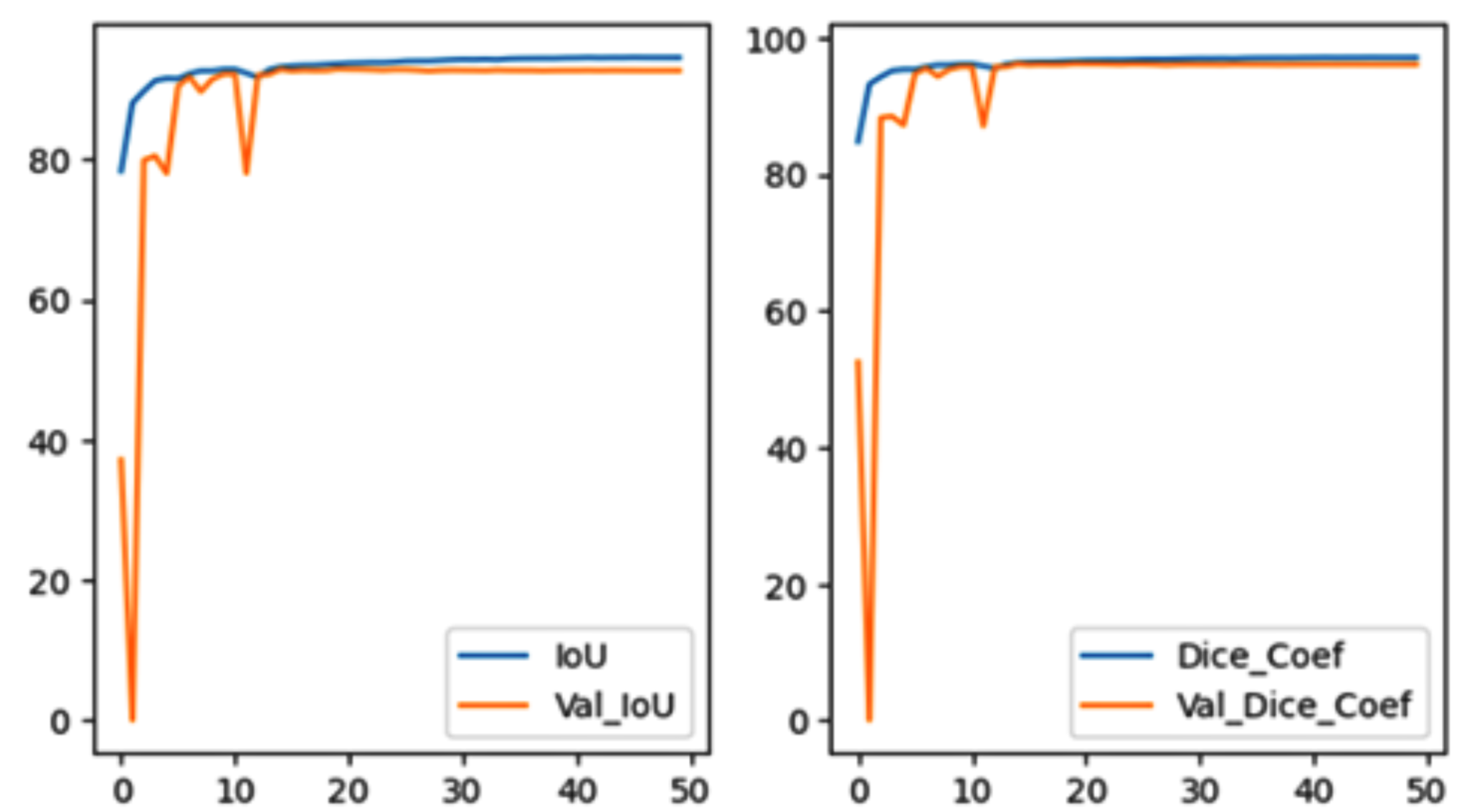

Supplement: Supplemental Information 7 [file peerj-cs-11-2700-s007.png]

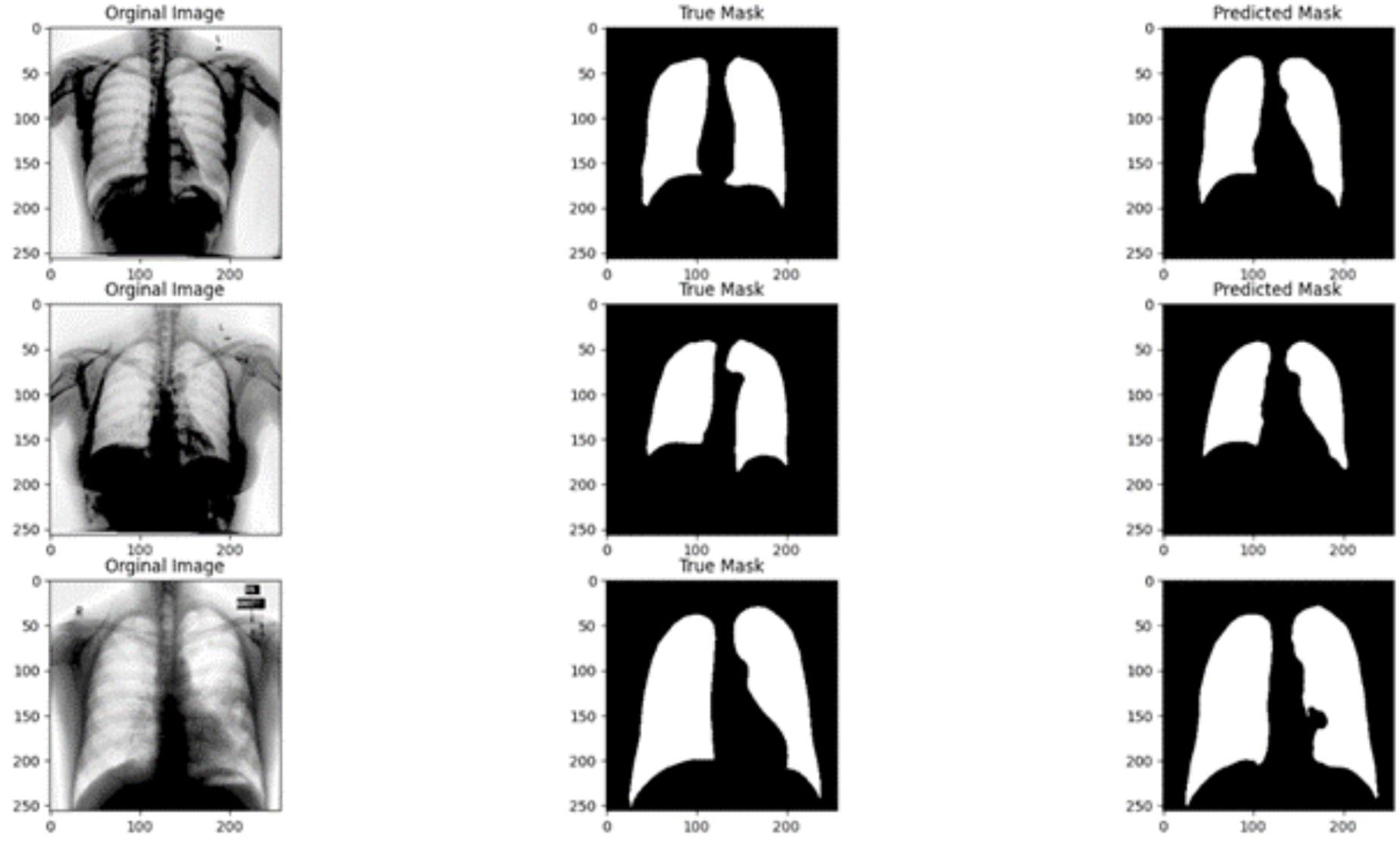

Supplement: Supplemental Information 8 [file peerj-cs-11-2700-s008.png]

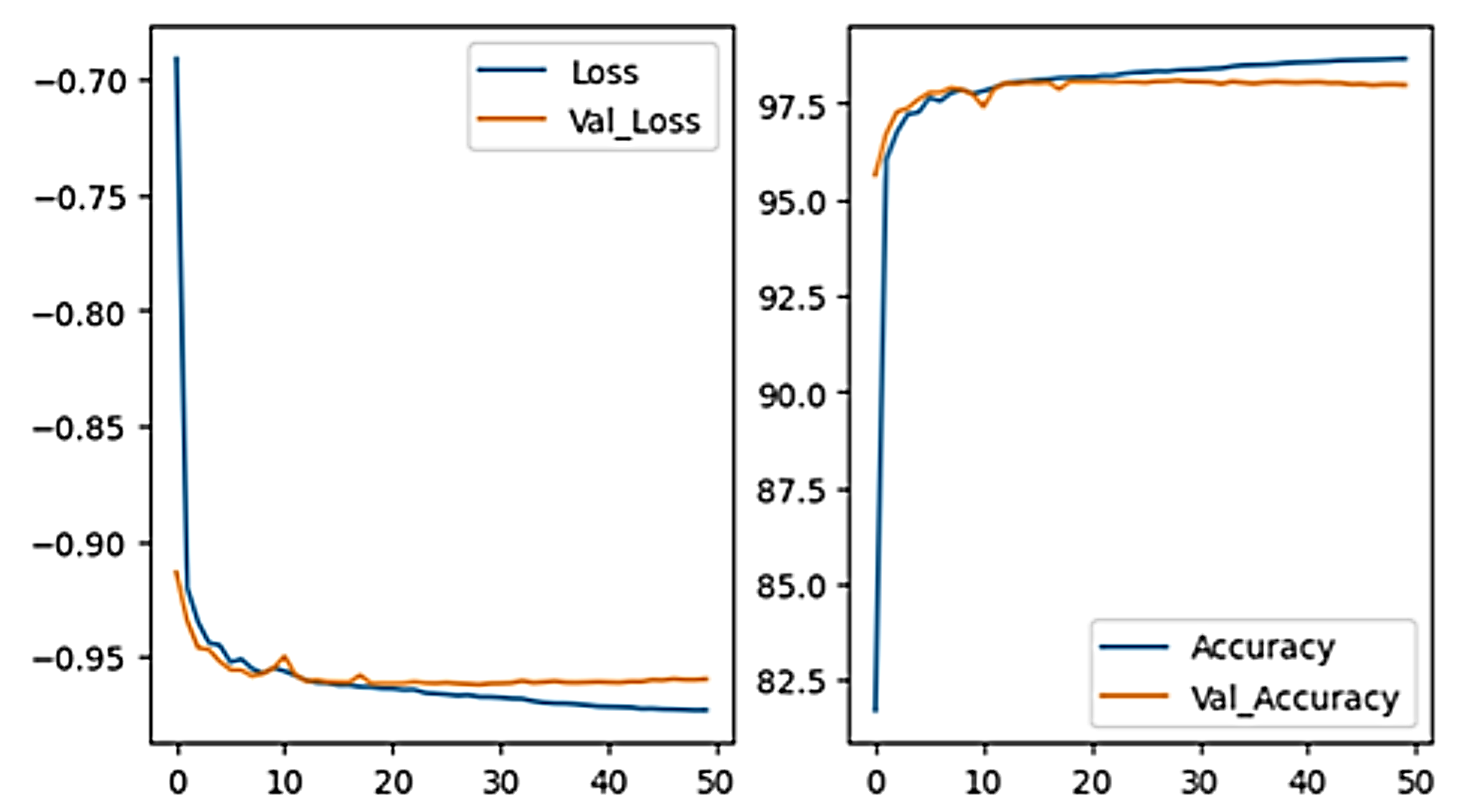

Supplement: Supplemental Information 9 [file peerj-cs-11-2700-s009.png]

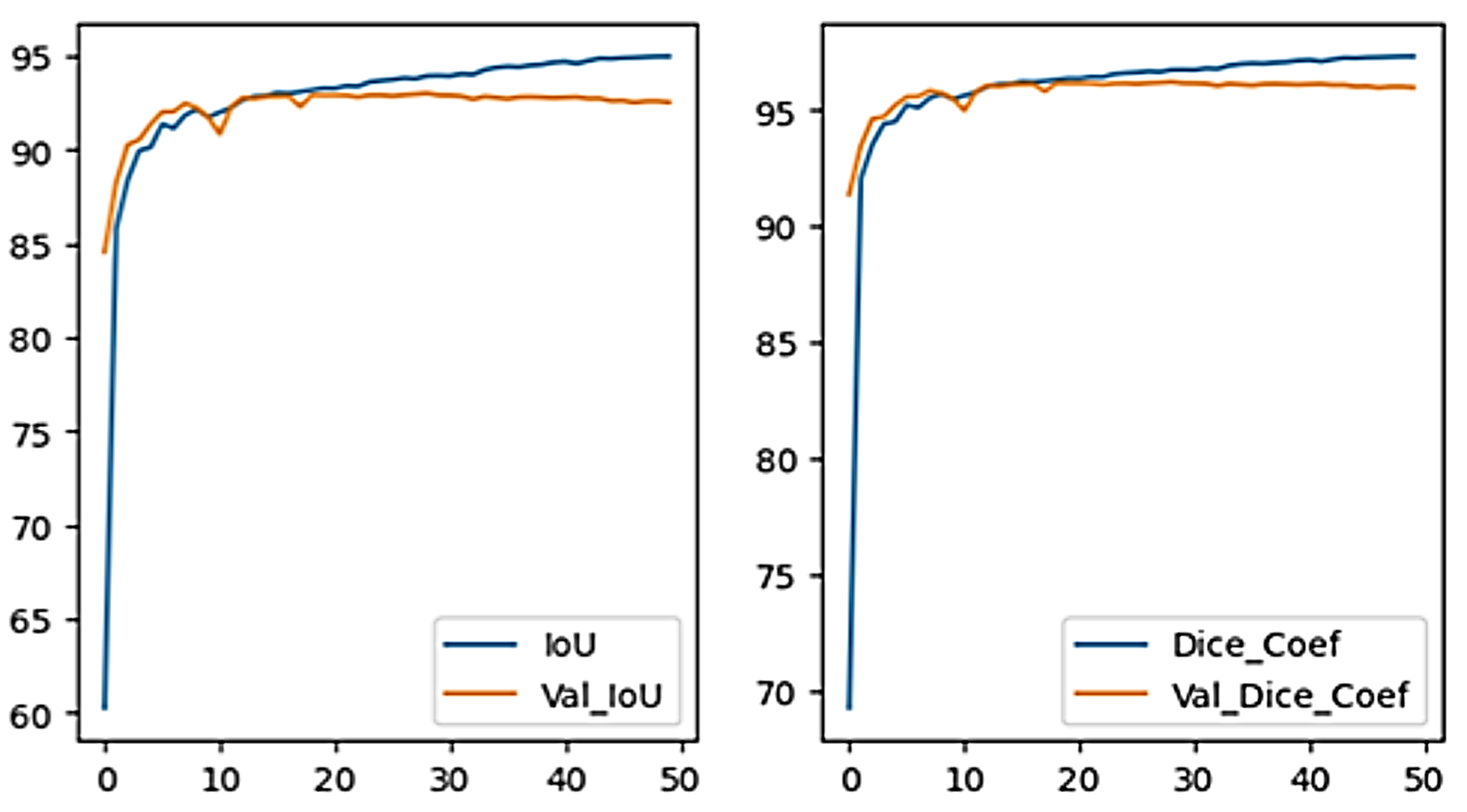

Supplement: Supplemental Information 10 [file peerj-cs-11-2700-s010.png]

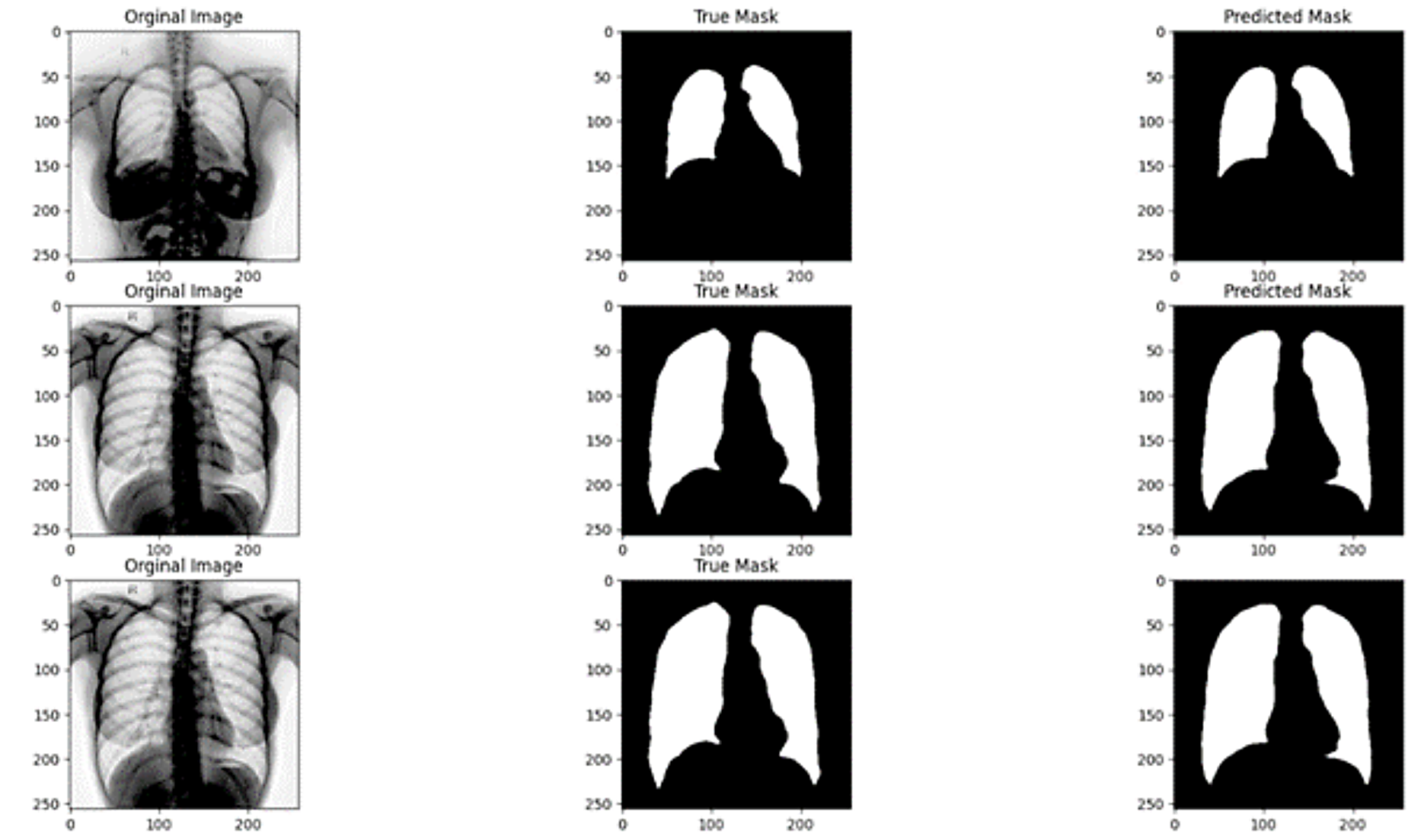

Supplement: Supplemental Information 11 [file peerj-cs-11-2700-s011.png]

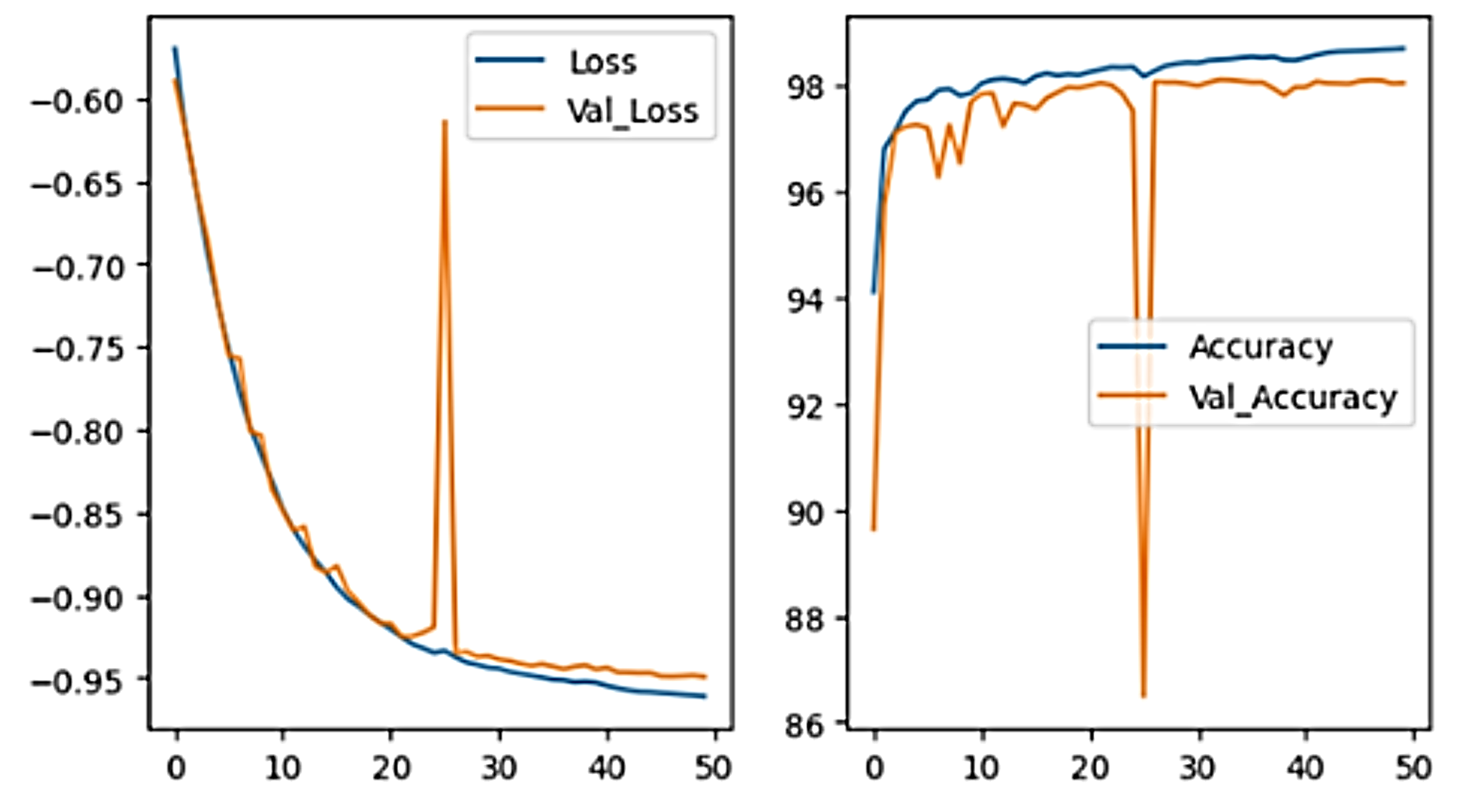

Supplement: Supplemental Information 12 [file peerj-cs-11-2700-s012.png]

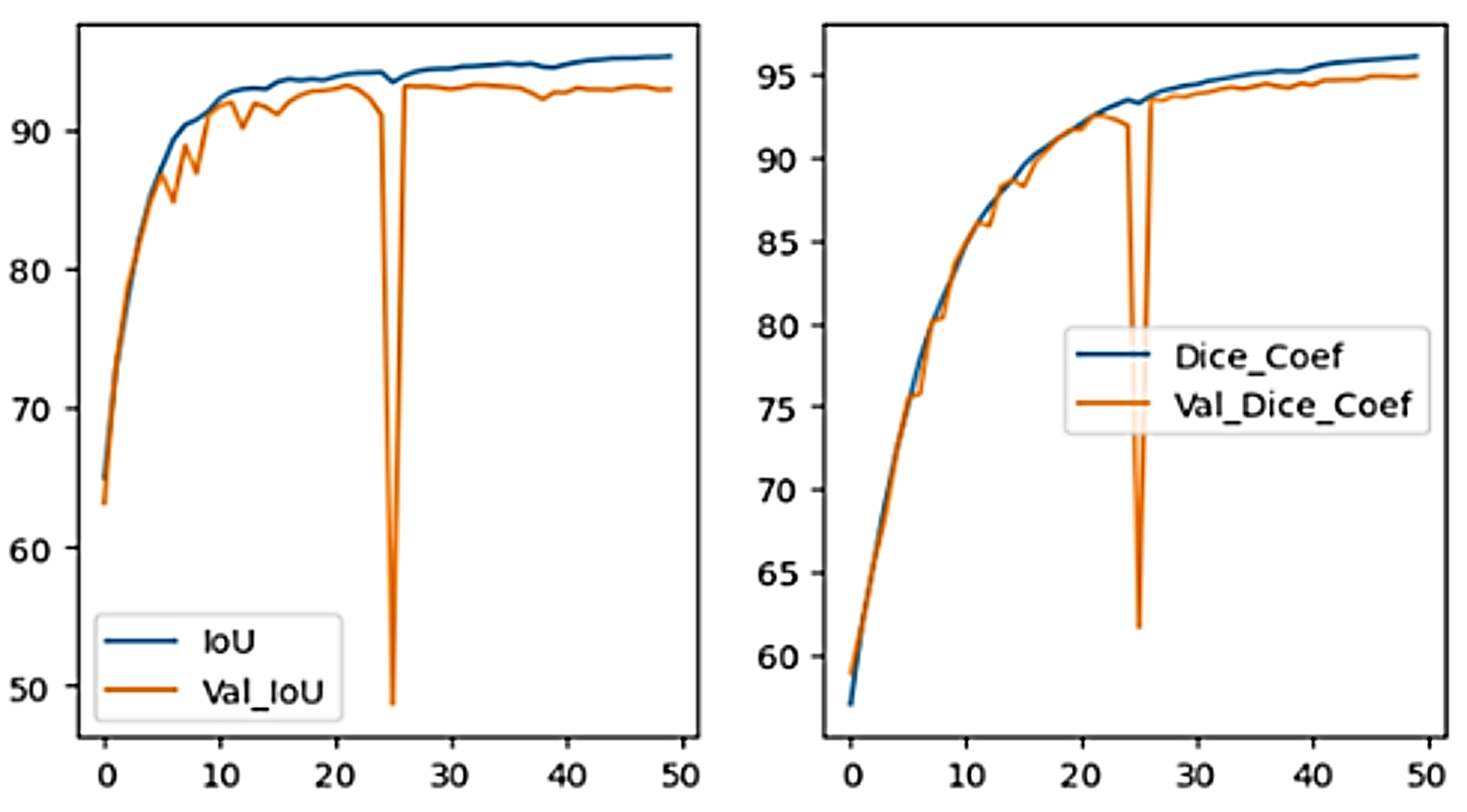

Supplement: Supplemental Information 13 [file peerj-cs-11-2700-s013.png]

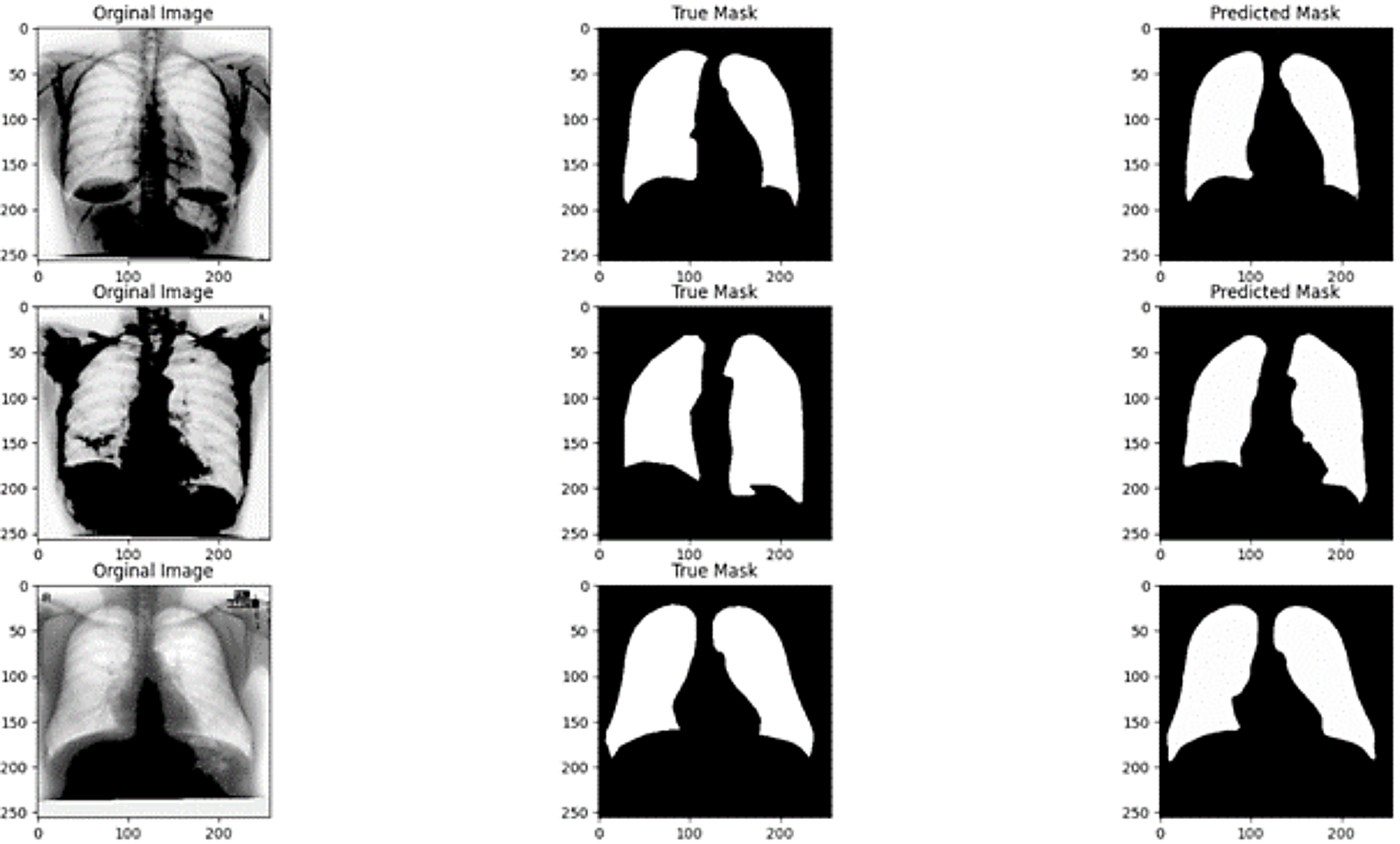

Supplement: Supplemental Information 14 [file peerj-cs-11-2700-s014.png]
